# Identification and quantification of *gyrA* variants in fluoroquinolone-resistant *Mycobacterium tuberculosis* in a MeltArray reaction

**DOI:** 10.1128/jcm.00146-25

**Published:** 2025-06-03

**Authors:** Chunxia Tu, Biyi Su, Yunfan Xiong, Zihan Xia, Chunlin Xiang, Yaoju Tan, Ye Xu, Qingge Li

**Affiliations:** 1 Engineering Research Centre of Molecular Diagnostics of the Ministry of Education, State Key Laboratory of Cellular Stress Biology, State Key Laboratory of Molecular Vaccinology and Molecular Diagnostics, School of Life Sciences, Faculty of Medicine and Life Sciences, Xiamen University 599936 , Xiamen, China; 2 Guangzhou Chest Hospital, Guangzhou, China; The University of North Carolina at Chapel Hill School of Medicine, Chapel Hill, North Carolina, USA

**Keywords:** *Mycobacterium tuberculosis*, FQs resistance, MeltArray, algorithm, heteroresistance, sputum, *gyrA*

## Abstract

Fluoroquinolones (FQs) resistance in *Mycobacterium tuberculosis* (MTB), primarily driven by mutations in the quinolone resistance-determining region (QRDR) of *gyrA*, poses a major concern in treating for multidrug-resistant tuberculosis (MDR-TB). These QRDR mutations are known to confer varying levels of resistance, leading to differences in treatment outcomes. Here, we introduced the MeltArray MTB/FQs assay, a multiplex PCR method that detected 11 *gyrA*-QRDR mutations and quantified their fractions via a polynomial regression algorithm-based formula, enabling mutation identification and quantification within 3 h in one reaction. This assay, with a limit of detection (LOD) of 50 copies/reaction, could identify mixtures containing 5% mutant gDNA across 50 to 50,000 copies/reaction. Clinical evaluation of 442 culture samples displayed 95.23% sensitivity and 99.32% specificity compared with phenotypic antimicrobial susceptibility testing (pAST). Evaluation of 121 paired sputum-culture samples revealed sensitivities of 90.32% in sputum samples and 95.24% in culture samples, both with specificities of 100%, when compared with pAST. Further evaluation of 285 sputum samples showed 93.75% positive percent agreement (PPA) and 98.10% negative percent agreement (NPA) in comparison with the MeltPro MTB/FQs kit (Zeesan Biotech, China). All mutant samples identified by MeltArray MTB/FQs but classified as susceptible by pAST or as wild type by MeltPro MTB/FQs were confirmed through Sanger sequencing and droplet digital PCR (ddPCR). The formula for predicting mutation fraction (MUT%) showed accuracy rates of 88.00%, 88.89%, and 83.33% in the training, validation, and test data sets, respectively, when compared with ddPCR results. Overall, MeltArray MTB/FQs assay offers an upgraded alternative for routine FQs resistance monitoring.

**IMPORTANCE:**

Rising FQs resistance has driven the spread of pre-extensively drug-resistant tuberculosis (pre-XDR-TB), challenging global tuberculosis (TB) control efforts. Conventional molecular assays for FQs resistance often cannot distinguish between low-level and high-level resistance mutations or detect low-fraction heteroresistant populations. In this study, we established a MeltArray MTB/FQs assay that can identify all the 11 critical mutations in the *gyrA*-QRDR with a LOD of 50 copies/reaction, enabling direct, culture-independent analysis of sputum samples. By using an algorithm to quantify mutations at levels as low as 5% in mixtures, MeltArray achieved both mutation identification and quantification within 3 h in a reaction, thus providing a powerful tool for early detection and precise management of pre-XDR-TB.

## INTRODUCTION

Fluoroquinolones (FQs), particularly moxifloxacin (MXF) and levofloxacin (LEV), are essential components for treating multidrug-resistant tuberculosis (MDR-TB) and rifampicin-resistant tuberculosis (RR-TB) (1, 2). Renowned for their potent bactericidal activity, excellent tissue penetration, and high oral bioavailability, FQs are integral to the World Health Organization (WHO) recommended shorter all-oral regimens (3). While their proper use has reduced morbidity and mortality from resistant strains, the alarming rise in FQs resistance is fueling the emergence of pre-extensively drug-resistant tuberculosis (pre-XDR-TB), defined as MDR-TB with additional resistance to any FQs, posing a critical threat to global tuberculosis control efforts (3–6).

The acquisition of resistance in *Mycobacterium tuberculosis* (MTB) is predominantly driven by mutations occurring within the quinolone resistance-determining region (QRDR) of *gyrA*, which encodes DNA gyrase, with alterations, especially at codons 90, 91, and 94, changing the quinolone binding pocket and causing cross-resistance to all FQs (7–10). One challenge in managing FQs resistance lies in the diverse phenotypic resistance levels associated with different *gyrA* mutation types, ranging from low-level, which is treatable with standard or higher doses, to high-level resistance, which typically requires exclusion from treatment regimens (8, 11–14). Moreover, while a single *gyrA* mutation in patients may result in low-level resistance, multiple co-occurring mutations can elevate resistance to a high level (10, 15). Consequently, the identification of mutation types and their co-occurrence is important for predicting resistance level.

Another challenge in managing FQs resistance is heteroresistance, which refers to the coexistence of both susceptible and resistant bacteria subpopulations within a sample (16). It has been reported that as many as 22% to 31% of patients with FQs-resistant infections were infected with both wild-type and QRDR mutant strains (17). According to the proportional method used in phenotypic antimicrobial susceptibility testing (pAST), an infection is classified as resistant if the proportion of resistant subpopulations exceeds 1% (18). Over time, resistant subpopulations may be enriched during inappropriate treatment, leading to full resistance (19). Consequently, heteroresistance serves as a warning for future resistance, highlighting the need for highly sensitive detection methods.

A variety of commercial molecular assays are available for rapid detection of FQs resistance; however, few can simultaneously identify specific mutation types and detect heteroresistance at high sensitivity. The WHO-endorsed GenoType MTBDRsl assay (Bruker-Hain Diagnostics, Germany) is capable of identifying only six *gyrA*-QRDR mutations and relies on complex post-PCR reverse hybridization procedures, which limit its broad implementation in routine clinical settings (20). The Xpert MTB/XDR assay (Cepheid, USA) employs three overlapping sloppy molecular beacons to identify three *gyrA-*QRDR mutations associated with low-level FQs resistance and distinguish them from those linked to high-level resistance (12). Moreover, Chakravorty et al. also applied such a melting temperature (*T_m_*)-coding strategy to identify known *gyrA*-QRDR mutations, but struggled to resolve co-occurring mutations, as multiple *T_m_* peaks cannot be deconvoluted into meaningful *T_m_* codes, resulting in samples often being identified as mixed DNA (12, 17). The MeltPro MTB/FQs assay (Zeesan Biotech, China), based on multi-color melting curve analysis, can detect the majority of *gyrA-*QRDR mutations in one reaction but is deficient in the ability to differentiate among them (21, 22). Furthermore, these assays either lack validation for heteroresistance detection or demonstrate limited sensitivity (20%–50%) (12, 17, 22). Thus, there is an unmet need for an alternative assay, which can not only identify all specific mutation types in *gyrA*-QRDR but also detect low-fraction heteroresistant mutations and even accurately determine their abundance levels.

We previously developed a multiplexed PCR platform termed MeltArray, which enabled the detection of multiple mutations in a single reaction and could identify heteroresistant mutation at levels as low as 5% (23). Hereby, we developed a MeltArray MTB/FQs assay to identify all the 11 *gyrA-*QRDR mutations in one reaction for evaluating FQs resistance. In addition to assessing its performance in detecting heteroresistance, we also explored its potential for quantifying the mutation fraction (MUT%). A comprehensive clinical evaluation was performed using 442 culture samples, 121 paired sputum-culture samples and 285 MTB-positive sputum samples, respectively.

## MATERIALS AND METHODS

### Design of the MeltArray MTB/FQs assay

**Fig 1:**
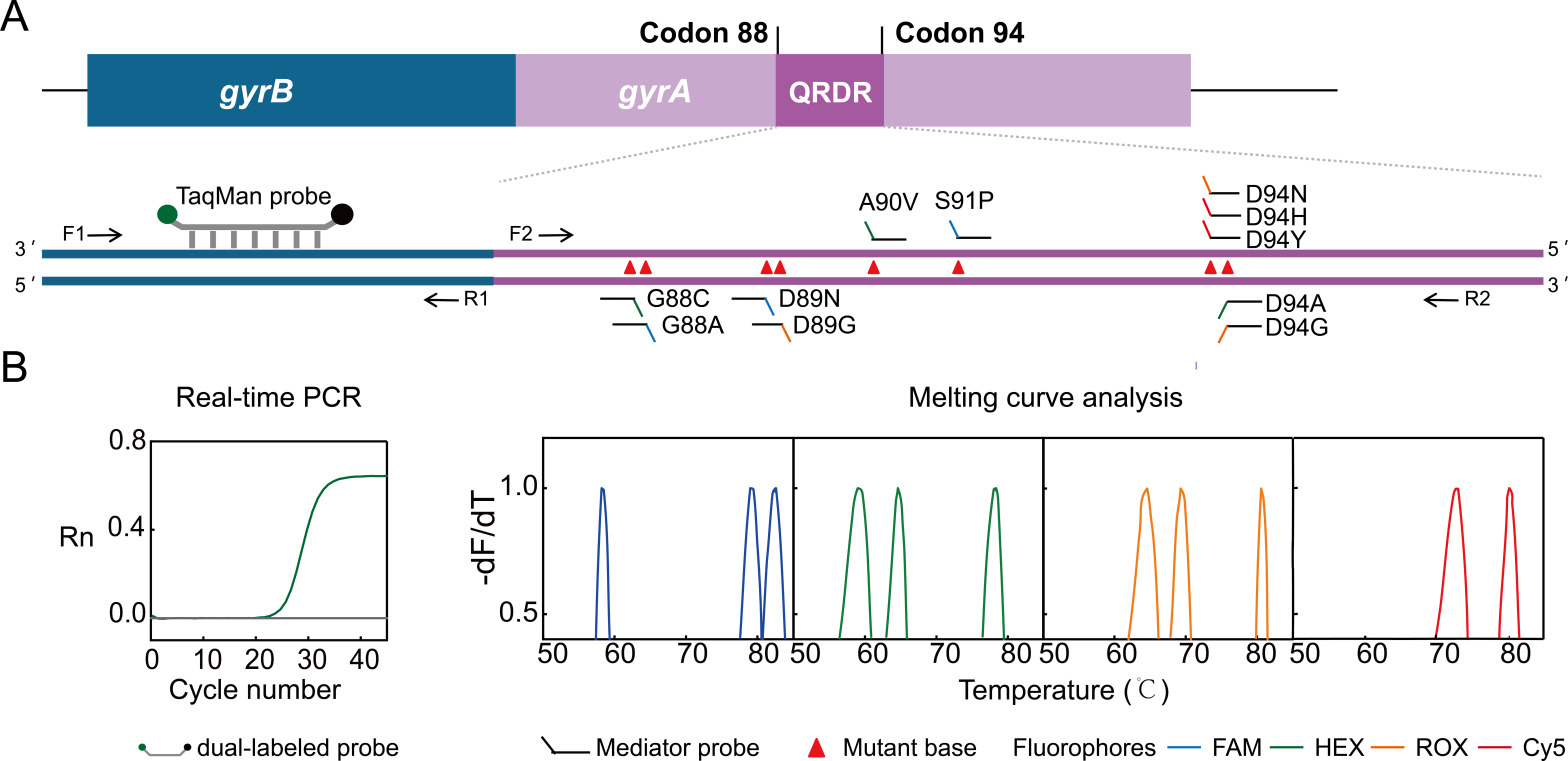
Schematic illustration of the MeltArray MTB/FQs assay. (**A**) The assay employs a pair of primers and a TaqMan probe to detect the conserved gene of *gyrB* for quantification, and an additional set of primers with 11 mediator probes to detect 11 resistance mutations in *gyrA*-QRDR. (**B**) The single-tube protocol enables simultaneous quantification of MTB abundance via Cq values from real-time PCR, and identification of the 11 key resistance mutations through specific "Fluorescence-*T_m_*" signatures obtained from melting curve analysis.

The MeltArray MTB/FQs assay aimed to identify all the 11 crucial *gyrA-*QRDR resistant mutations, i.e., G88A, G88C, D89N, D89G, A90V, S91P, D94G, D94A, D94H, D94N, and D94Y, in line with the WHO’s second edition of the “*Catalogue of Mutations in MTB Related to Drug Resistance*” (24). Based on the working principle of MeltArray, two sets of primers and probes were designed. The first set was used to detect the *gyrB* gene in a real-time PCR assay. The generated Cq value during the denaturation stage of PCR was used to estimate the abundance of MTB. The second set, containing a common pair of primers and 11 mediator probes, was used to detect the 11 *gyrA-*QRDR mutations in the melting curve analysis mode after PCR. The *T_m_* value generated in the corresponding fluorometric channel was used to indicate the presence of mutation (Fig. 1A).

### Workflow of the MeltArray MTB/FQs assay

The MeltArray MTB/FQs assay was carried out using the SLAN-96 thermocycler (Hongshi, China). The reaction was run in a 25 µL solution containing 10 × PCR buffer (Zeesan Biotech, China), 6 mM MgCl_2_, 0.6 mM dNTPs, 3.0 U of Taq01 DNA polymerase, and 5 µL of genomic DNA (gDNA) template. Each reaction contained two pairs of primers, 11 mutation-specific mediator probes, one *gyrB*-specific TaqMan probe, and seven universal molecular beacon reporters (sequences and concentrations are listed in Table S1). The PCR program was conducted as follows: 95°C for 10 min; 95°C for 25 s, 60°C for 1 min for 45 cycles, with fluorescence collected in the HEX channel; 35°C for 30 min, 45°C for 2 min; and then a temperature increase from 45°C to 95°C at 0.16°C/s with fluorescence signals collected in the FAM, HEX, ROX and Cy5 channels.

### Analytical evaluation

The limit of detection (LOD) of MeltArray MTB/FQs assay was assessed using 11 distinct plasmids containing *gyrA* mutations and one plasmid containing *gyrB* conserved sequence at four concentrations: 5,000, 500, 50, and 5 copies/reaction. Twenty replicates were tested per concentration to determine LOD, defined as the lowest concentration with ≥ 95% positive detection (25). The lowest concentration that yielded accurate results for the majority of *gyrA*-QRDR mutations was used to establish the cut-off of quantification cycle (Cq), defined as the average Cq from the 20 tests of the *gyrB* plasmid, minus two fold standard deviation (SD) (26).

To further assess our assay’s performance in clinically relevant matrices, we assessed the LOD using intact MTB bacteria in both culture specimens and sputum specimens. For culture-based testing, serial dilutions of the wild-type reference strain H37Rv and a *gyrA* A90V mutant strain were prepared in saline at final concentrations of 20,000, 2,000, 200, and 20 bacilli/mL. gDNA was extracted using the Lab Aid-824S system (Zeesan Biotech, Xiamen, China) following the manufacturer’s instructions. For sputum-based testing, MTB-negative clinical specimens were spiked with defined concentrations of either wild-type or mutant strains and processed similarly to the culture specimens, with the addition of a sputum liquefaction step prior to DNA extraction. The LOD was defined as the lowest bacterial concentration at which ≥ 95% of replicates (*n* = 20) yielded a positive result.

The LOD for heteroresistance (LOD-HR) was evaluated using mixtures of wild-type and 11 mutant plasmids prepared at MUT% values of 1%, 5%, 10%, 20%, 50%, and 100%. LOD-HR of each mutation was tested at four concentrations of 50,000, 5,000, 500, and 50 copies/reaction, with three parallel replicates tested at each MUT%.

To evaluate *T_m_* reproducibility, three parallel replicates containing 50 copies/reaction of plasmid DNA were tested for each mutation. The experiments were repeated on three different SLAN-96 thermocyclers at three separate times, generating nine *T_m_* values per target, from which the average *T_m_* and SD were calculated. Specificity was assessed by gDNA templates prepared from MTB reference strain H37Rv, 24 nontuberculous mycobacteria (NTM), and 18 other non-mycobacterial strains (Table S2).

Eleven plasmids each containing distinct *gyrA*-QRDR point mutation and one plasmid containing both a segment of *gyrB* conserved sequence and a segment of *gyrA* wild-type sequence (defined as internal positive control [IPC]) were synthesized by Tsingke Biotechnology Co., Ltd. (Xiamen, China). The gDNA of MTB reference strain H37Rv was provided by the National Institutes for Food and Drug Control (NIFDC). gDNAs from 24 NTM and 18 common respiratory non-mycobacterial strains were stored in our laboratory. The concentrations of gDNA were measured using an ND-1000 spectrophotometer (NanoDrop Technologies, Wilmington, DE, USA).

### Clinical evaluation

**Fig 2:**
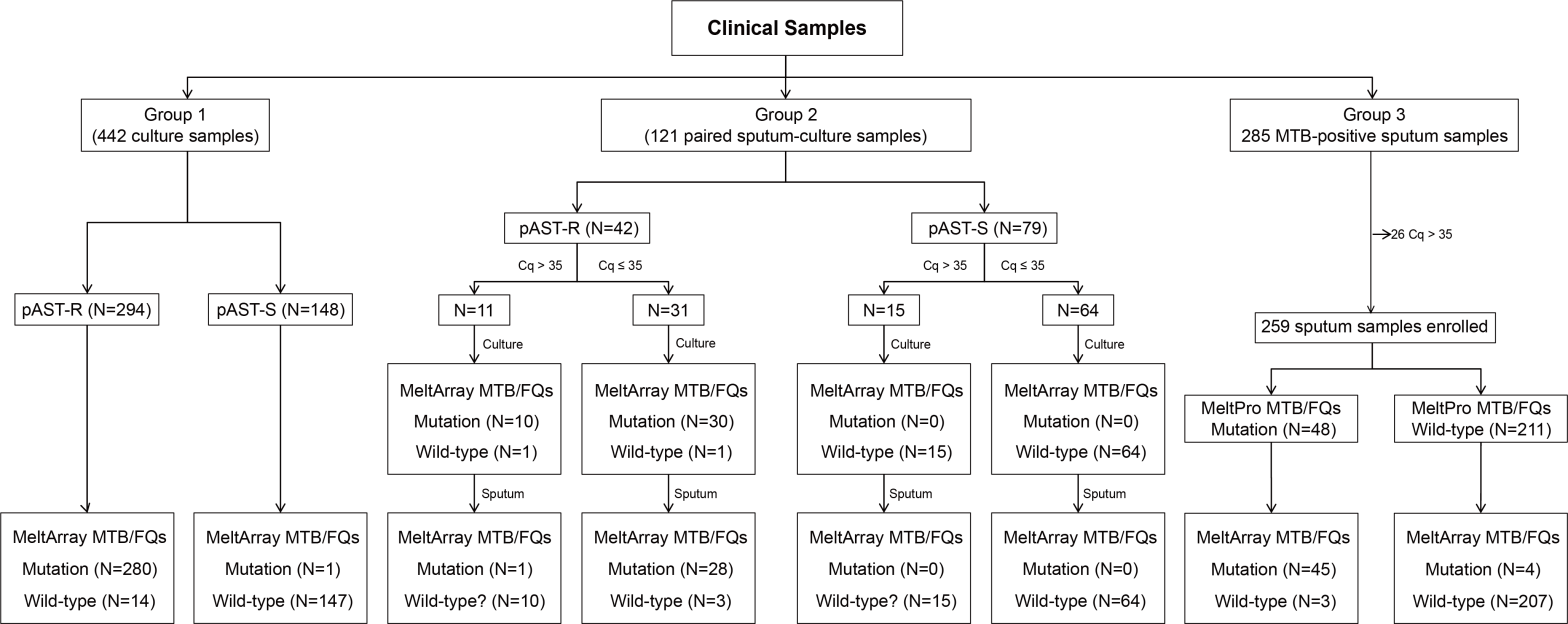
Clinical performance of the MeltArray MTB/FQs assay across three groups. “Wild-type?” indicates that the assay cannot confirm a wild-type genotype when no mutation is detected and the cq value exceeds 35. pAST-R and pAST-S refer to phenotypic antimicrobial susceptibility testing-resistant and -sensitive results, respectively, representing phenotypic resistance or susceptibility to FQs.

Three groups of samples were collected for clinical evaluation (Fig. 2). Group 1 comprised 442 archived MTB gDNA extracted from culture isolates stored in our laboratory, and they were used to validate the accuracy of MeltArray MTB/FQs in identifying mutations and assess clinical diagnostic performance. Group 2 included 121 paired sputum-culture samples from Guangzhou Chest Hospital (Guangzhou, Guangdong, China). Specifically, each paired sputum-culture sample in Group 2 was originated from a single sputum sample, which was divided into two portions: one for mycobacterial culture by MGIT960 liquid culture system (Becton, Dickinson, MD, USA) (see File S1 for details) and pAST, and the other retained for direct analysis. Only culture-positive samples were included in the subsequent analysis. The MeltArray MTB/FQs assay was performed on both the original sputum samples and their corresponding cultured isolates, and the results were compared against pAST, which served as the reference standard. Group 3 consisted of 285 sputum samples from Guangzhou Chest Hospital to assess the ability of MeltArray MTB/FQs for rapid testing. The sputum samples from Group 3 were identified as MTB-positive using a commercial MTB real-time PCR test kit (Zeesan Biotech, China). Screening of FQs resistance in sputum samples from Group 3 were carried out using a commercial MeltPro MTB/FQs kit (Zeesan Biotech). The detailed procedures for the above two kits were provided in File S2 and S3. The clinical evaluation study was reviewed and approved by the Institutional Review Board of Guangzhou Chest Hospital. Informed consent was obtained from all the patients.

To ensure objectivity, an independent, blinded review process was implemented. gDNA samples were recoded by two supervisors (B.S. and Y.T.), who also oversaw the collection of clinical data. The recorded samples were analyzed by three researchers (C.T., C.X., and Z.X.) without prior access to pAST results. The detection outcomes were independently verified by two additional researchers (Q.L. and Y.X.), who conducted the concordance assessments and performed the statistical analysis.

The MTB gDNA from both culture samples and sputum samples was extracted according to previously described protocols (27) and stored at −80°C before use. Briefly, culture samples (~300 µL) were directly subjected to gDNA extraction, whereas sputum samples (~500 µL) were first liquefied prior to extraction. gDNA extraction was performed using the Lab Aid-824S system (Zeesan Biotech) following the manufacturer’s instructions.

### Phenotypic antimicrobial susceptibility testing (pAST)

pAST of the culture samples (Group 1 and Group 2) was performed using the standard proportion method on Löwenstein–Jensen medium. Critical concentrations of 1.0 µg/mL for MXF and 2.0 µg/mL for LEV were applied in accordance with WHO recommendations (28).

### Polynomial regression algorithm-based formula for predicting MUT%

The polynomial regression algorithm was established using the NumPy and SciPy libraries based on Python. The polyfit function in NumPy was used for initial polynomial fitting, and parameter refinement was conducted using optimization tools from SciPy. We then employed root mean square error (RMSE) as the loss function to assess model performance and minimize prediction error following $\text{ RMSE }=\sqrt{\frac{1}{N}\sum_{i=1}^{N}{\left({\text{ observed }}_{i}-{\text{ predicted }}_{i}\right)}^{2}}$, where observed values were derived from ddPCR-measured MUT%, representing actual quantitative data, whereas predicted values were generated by the polynomial regression model as estimated MUT%.

Taking D94G as an example, we analyzed the relationship between melting peak height (Rm) and MUT% across five DNA concentrations. Using a polynomial regression algorithm, we derived a formula for calculating MUT%: $\frac{Rm+(6.11E-11)C^{1}-(5.35E-9)C^{2}+(1.17E-13)C^{3}-(2.12E-19)C^{4}}{(-4.22E-11)C^{1}+(3.69E-9)C^{2}-(7.22E-14)C^{3}+(8.96E-20)C^{4}}\cdot k$, where $C=10^{\frac{\left(Cq-37.62\right)}{\left(-3.48\right)}}$, and *k* is a correction factor introduced to adjust for the linear bias between observed and predicted values. This factor is iteratively optimized over 5,000 iterations using the gradient descent method.

Of the 43 D94G heteroresistant mutations in Group 1 validated by ddPCR, 25 were randomly selected as the training set to optimize the parameter *k* and model accuracy, while the remaining 18 were used as the validation set. To assess whether the MUT% estimated by the polynomial regression-based formula demonstrated equivalent detection accuracy to that obtained by ddPCR, we conducted a two-sided equivalence test with a predefined margin of 10%. If the difference between the formula-derived MUT% and the ddPCR result fell within this margin, the prediction was deemed accurate (29).

### Sanger sequencing

Sanger sequencing was used to verify the accuracy of mutation detection. The sequencing primers and amplification procedures were in accordance with the previous study (22). Amplification products were purified and sequenced by Sangon Biotech Co., Ltd. (Shanghai, China).

### ddPCR MTB/FQs assay

ddPCR assay was used to validate discrepancies between MeltArray MTB/FQs and Sanger sequencing and also to quantify MUT%. ddPCR was performed in a 30 µL mixture containing 15 µL of 2 × SuperMix (TargetingOne, Beijing, China), 0.96 µM primers (forward, 5′-CACGCCAAGTCGGCCCGGT-3′ and reverse, 5′-AGCGGGTAGCGCAGCGA-3′), 0.75 µM probes (5′-(FAM)-ATCTACGACACCTGGTGCGC-(BHQ1)-3′ and 5′-(HEX)-AGATCGACGCGTCGCCGT-(BHQ1)-3′) (note: the underlined nucleotides indicate locked nucleic acids [LNAs]), and 5 µL of gDNA template. Droplet generation, micro-emulstion amplification, and droplet detection were performed using the TargetingOne ddPCR system (TargetingOne Inc).

### Statistical analysis

Clinical diagnostic performance, including sensitivity, specificity, positive percent agreement (PPA), and negative percent agreement (NPA), was calculated using two reference standards. pAST results were regarded as the reference for the 442 culture samples (Group 1) and 121 paired sputum-culture samples (Group 2), and the MeltPro MTB/FQs results were used as the reference for the 285 MTB-positive sputum samples (Group 3). Clinical evaluation results were analyzed using the OpenEpi online tool (https://www.openepi.com/) with 95% confidence intervals. The kappa value was calculated by SPSS 23.0 (IBM, Armonk, NY, USA), with kappa > 0.75 indicating excellent consistency, a kappa < 0.45 indicating poor consistency, and kappa = 0.45-0.75 indicating moderate consistency (30). Two-sided tests were performed, with *P* < 0.05 considered statistically significant. Figures were prepared and formatted using Origin 2019 (OriginLab, Northampton, MA, USA) and Adobe Illustrator CC 2019 (Adobe Systems Incorporated, San Jose, CA, USA).

## RESULTS

### Establishment of MeltArray MTB/FQs assay

The designed MeltArray MTB/FQs assay was simply a duplex PCR, yet it was a bit complex when considering its inclusion of 11 single point mutation-specific probes, in particular, most of which were closely overlapped. Candidate mediator probes based on hybridization (D94H, D94Y, D94A, and D94N) and site-specific endonuclease (G88C, G88A, D89N, D89G, A90V, S91P, and D94G), targeting both forward and reverse strands, were designed for each mutation. Only those that yielded the largest signal-to-noise ratio were chosen. The final 11 mediator probe sequences are shown in Table S1. Special attention was paid to the polymorphic D95 site and the mutations located in the overlapping probe-binding regions, where degenerate bases (i.e., multiple possible nucleotides at a single position) were introduced to avoid reduced probe binding efficiency. Mutation distribution in the four fluorometric channels was also optimized for better resolution of adjacent melting peaks and prevention of nonspecific melting peaks. The final MeltArray MTB/FQs assay included the real-time PCR detection of *gyrB* in the HEX channel and melting curve analysis of 11 mutations in FAM, HEX, ROX, and Cy5 channels. Representative results using different plasmids showed that both real-time PCR detection and melting curve analysis produced the expected results (Fig. 1B). Using the thermocycling program described in the Materials and Methods section, the MeltArray assay was completed within 2.5 h following the addition of gDNA template into the reaction.

### Analytical performance of MeltArray MTB/FQs assay

The LOD for MeltArray MTB/FQs assay was 50 copies/reaction by using the plasmid DNA (Fig. S1A), with a defined Cq cut-off of 35 (95% CI: 35.95-36.53; Fig. S1B). A linear correlation could be seen (R² = 0.99987) between Cq value and the logarithm of template concentration ranged from 10^0^ to 10^4^ copies/μL (Fig. S1B). The LOD for intact MTB in culture specimens and sputum specimens was further assessed. As shown in Fig. S2, the LOD was 200 bacilli/mL for both the wild-type and the *gyrA* A90V mutant in culture specimens, and 200 bacilli/mL for the wild-type strain and 500 bacilli/mL for the *gyrA* A90V mutant in sputum specimens. Specificity study demonstrated that neither amplification curve nor melting curve was observed for all the 24 NTM and 18 non-mycobacterial strains (Table S2). *T_m_* reproducibility study showed that 3SD < 0.50°C and coefficient variation (CV) ≤ 0.20%, with Δ*T_m_* > 3.5°C between adjacent peaks, ensuring unambiguous separation (Table S3). The interpretation threshold for each mutation was established to ensure consistent mutation analysis (Table S3).

The LOD-HR study showed that, at 50,000 copies/reaction, all 11 mutations were detectable at 5% mutant proportion. At 5,000 copies/reaction, most mutations retained a 5% detectability, except for D94H and D94Y, which required 10%. When at 500 copies/reaction, D89N, G88A, S91P, A90V, D89G, and D94N were detectable at 5%, while the remaining mutations required 10%. Further analysis at 50 copies/reaction showed that S91P and D94N maintained a 5% detection threshold, while other mutations dropped to 10% to 20% (Fig. S1A).

### Clinical evaluation of the MeltArray MTB/FQs assay using 442 culture samples (Group 1)

**Fig 3:**
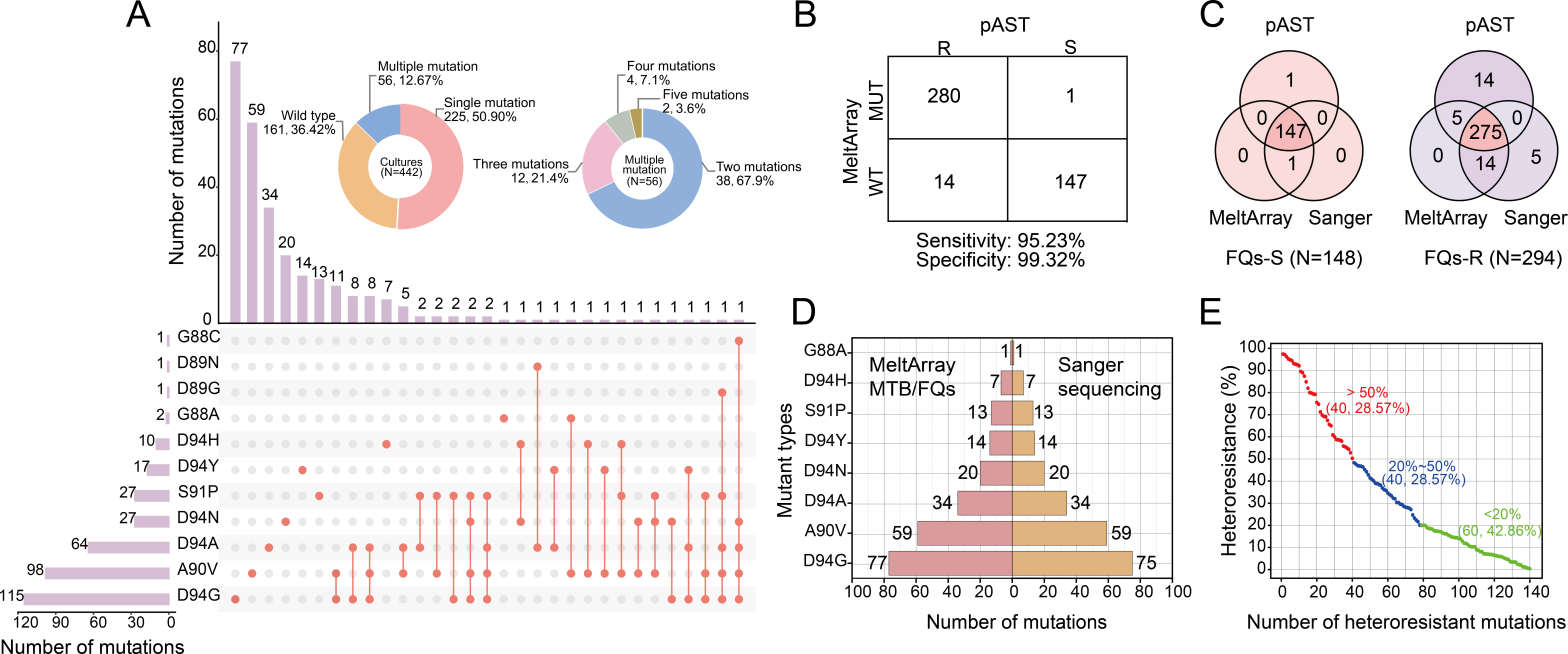
Clinical evaluation of the MeltArray MTB/FQs assay in 442 culture-positive samples from Group 1. (**A**) Overview of mutations detected. The upper pie charts depict the proportions of single-mutation, multiple-mutation, and wild-type samples, along with the distribution of mutation counts within the multiple-mutation group. The lower UpSet plot illustrates the frequencies and co-occurrence patterns of *gyrA* mutations. Red dots in the matrix indicate individual mutations, and red lines show co-occurring mutations within the same sample. The left purple bars represent the overall frequency of each mutation, while top purple bars display the frequencies of specific mutation patterns. (**B**) A 2 × 2 contingency table comparing the performance of MeltArray assay with pAST. (**C**) Comparison of results obtained from pAST, MeltArray, and Sanger sequencing in the 442 culture samples. (**D**) Comparison of the mutation identification results between the MeltArray assay and Sanger sequencing in 225 single mutation samples. Each bar represents a specific mutation type (y-axis), and the length of the bar (x-axis) indicates the number of samples in which that mutation was detected by either method. (**E**) Distribution of 140 heteroresistant mutation fractions across 56 multi-mutant samples. Green dots indicate low-fraction mutations (< 20%), blue dots represent intermediate-fraction mutations (20%-50%), and red dots indicate high-fraction mutations (> 50%).

A total of 31 distinct mutation formats (defined as specific mutation types and their combinations) were identified, including eight single-mutant and 23 multi-mutant. The number of mutations in multi-mutant samples ranged from 2 to 5 (Fig. 3A). Of the total, 225 (50.90%) were classified as single-mutant, 56 (12.67%) as multi-mutant, and 161 (36.42%) as wild-type. While single-mutant samples were predominant, all 11 target mutations were detected in multi-mutant samples. Notably, three rare mutations D89G, D89N, and G88C were found exclusively in multi-mutant samples (Fig. 3A).

Using the pAST results as the reference, the MeltArray assay demonstrated a clinical sensitivity of 95.23% and specificity of 99.32% (Fig. 3B). Discrepancies were observed in 15 samples: 14 samples were classified as wild type by MeltArray but were resistant by pAST, while one sample was classified as mutant by MeltArray but susceptible by pAST. These discrepancies were fully resolved by Sanger sequencing, which showed complete agreement with the MeltArray results regarding mutant status (classified as either mutant or wild type). Additionally, Sanger sequencing of the *gyrB* gene spanning codons 461 to 499 identified two D461N mutations (Table S4), which were outside the scope of the MeltArray assay. Furthermore, an additional five samples, classified as wild type by Sanger sequencing but resistant by pAST, were all identified as mutant by MeltArray (Fig. 3C). This finding highlighted the higher sensitivity of the MeltArray assay compared with Sanger sequencing (95.23% vs 93.54%), although both methods showed identical specificity (99.32%; Fig. 3B; Fig. S3).

Next, we compared the mutation format of each sample between the MeltArray and Sanger sequencing. For the 225 single-mutant samples, their concordance rate was 99.11% (223/225; Fig. 3D). For the 56 multi-mutant samples, their concordance reduced to 48.21% (27/56; Table S5). ddPCR analysis of these 56 multi-mutant samples revealed 140 distinct heteroresistance mutations, mutation fraction ranging from 0.92% to 97.40% (Fig. 3E). All discrepant mutation formats between the MeltArray and Sanger sequencing were due to additional mutations detected by MeltArray but missed by Sanger sequencing (Table S6). With the exception of two samples that contained a single minor mutation with a heteroresistance level (< 2%) below LOD-HR, all inconsistent results were perfectly matched between the MeltArray assay and ddPCR (Table S6). These findings suggested that the MeltArray assay outperformed Sanger sequencing in detecting low-fraction heteroresistant mutations.

### Clinical evaluation of MeltArray MTB/FQs assay using 121 paired sputum-culture samples (Group 2)

**Fig 4:**
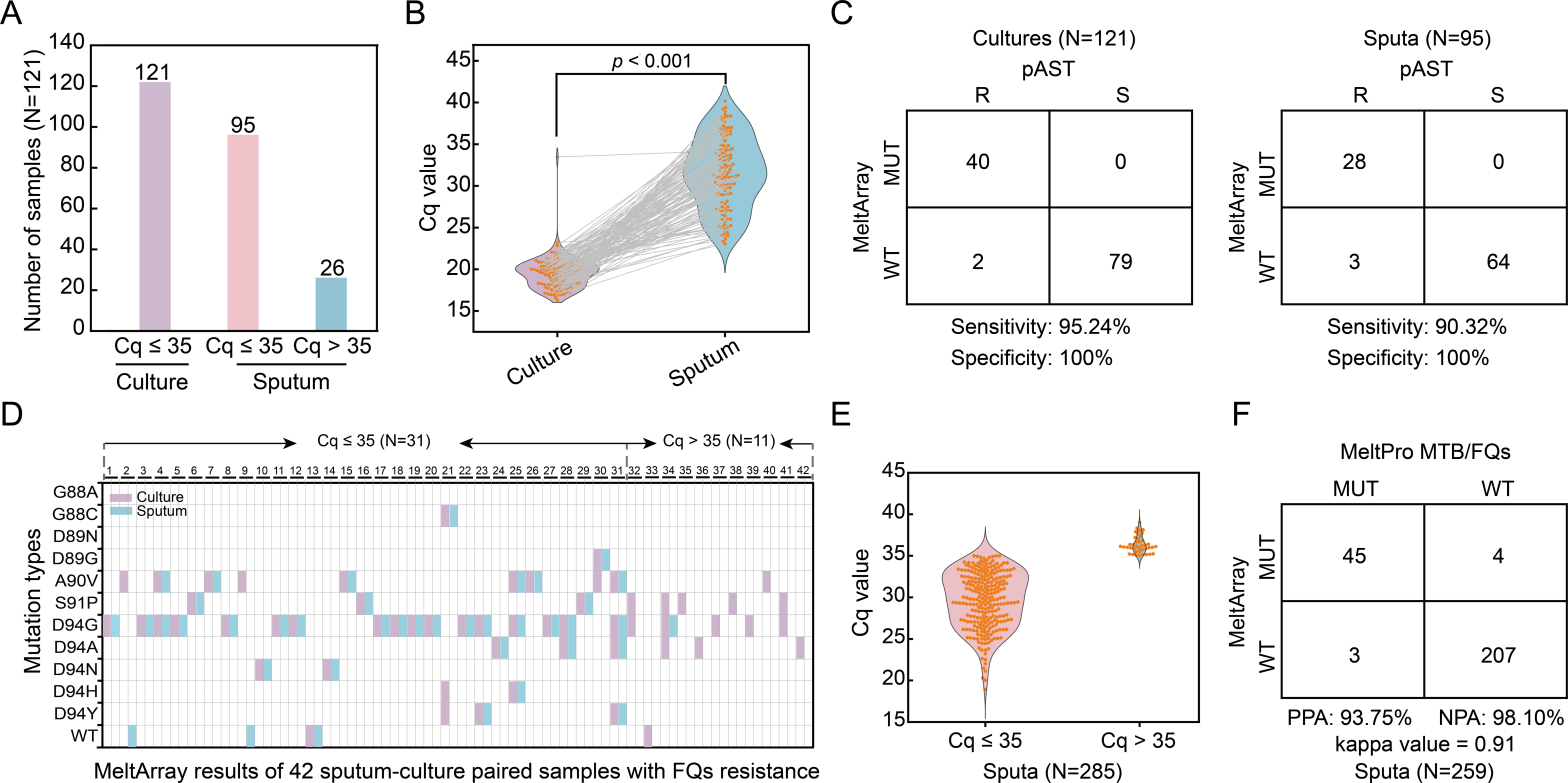
Clinical evaluation of the MeltArray MTB/FQs assay in 121 paired sputum-culture samples from Group 2 and 285 MTB-positive sputum samples from Group 3. (**A**) Classification of the 121 paired sputum-culture samples using a Cq value threshold of 35. (**B**) Comparison of Cq values between paired sputum-culture samples, with each connected by a gray line. (**C**) A 2 × 2 contingency table comparing the performance of MeltArray assay with pAST in samples with Cq ≤ 35 from group 2. (**D**) Paired MeltArray results for the 42 FQs-resistant samples in Group 2. (**E**) Classification of 285 MTB-positive sputum samples using a Cq value threshold of 35. (**F**) A 2 × 2 contingency table comparing the performance of MeltArray assay with MeltPro assay in samples with Cq ≤ 35 from group 3. PPA, positive percent agreement; NPA, negative percent agreement.

Among the 121 paired sputum-culture samples, pAST revealed that 42 (34.71%) were FQs-resistant, and 79 (65.29%) were FQs-susceptible (Fig. 2). MeltArray MTB/FQs assay showed that all liquid culture samples passed quality control (Cq ≤ 35), with average Cq values of 19.59 (Fig. 4A and B), yielding a clinical sensitivity of 95.24% and a specificity of 100% (Fig. 4C). Sanger sequencing also provided valid results for all liquid culture samples, with a sensitivity of 90.48% and specificity of 100% (Fig. S4A). For the paired sputum samples, the MeltArray assay generated valid results for 95 (78.51%) samples (Fig. 4A), achieving a sensitivity of 90.32% and specificity of 100% (Fig. 4C). Sanger sequencing produced valid results for 80 (66.12%) sputum samples, yielding a sensitivity of 88.89% and specificity of 100% (Fig. S4B). Detailed sequencing results can be found in Fig. S5. Impressively, mutations were only detected in one sputum sample with a Cq value > 35 (Fig. 4D), underscoring the appropriateness of the Cq threshold set for MeltArray assay. We further compared the Cq values obtained from Group 2 samples with their corresponding smear grades. As expected, an inverse correlation was observed between smear grade and Cq value (Fig. S6). Notably, a subset of both smear-positive and -negative samples still yielded successful MeltArray results.

We then evaluated the consistency between culture and paired sputum samples with regard to mutant status and mutation format. Among the 42 paired sputum-culture samples resistant to FQs, 31 (73.81%) passed quality control (Cq ≤ 35), and of these, 29 (93.55%) showed consistent mutant status, while two sputum samples yielded wild-type results (Fig. 4D). Regarding mutation formats of the 29 pairs of samples with consistent mutant status, 27 (93.10%) shared identical mutation formats, and two pairs of multi-mutant samples exhibited different mutation formats. Specifically, three mutations (D94H plus D94Y and A90V) were undetected in two sputum samples (Fig. 4D).

### Clinical evaluation of MeltArray MTB/FQs assay in 285 MTB-positive sputum samples (Group 3)

In this group, 259 (90.88%) of the samples had Cq ≤ 35, qualifying them for further testing (Fig. 4E). These samples were tested using both the MeltArray and MeltPro assays. The PPA and NPA for the MeltArray were 93.75% and 98.10%, respectively, with a kappa of 0.91, indicating a high level of agreement between the two methods (Fig. 4F). The seven discrepant samples were analyzed using ddPCR as Sanger sequencing failed to yield any valid results. Among the four samples that were detected as mutant by MeltArray but as wild type by MeltPro, ddPCR analysis confirmed the MeltArray results. In contrast, for the three samples identified as wild type by MeltArray but as mutant by MeltPro, ddPCR confirmed that two were wild type, while the third sample had a D94G mutation (Table S7). Together, these results demonstrated that the MeltArray assay was more accurate than the MeltPro assay for mutation detection in this group of MTB-positive sputum samples.

### Polynomial regression algorithm-based formula for predicting MUT%

During the evaluation of LOD-HR, we observed that the Rm of melting peaks varied with MUT% in different template concentrations (Fig. S7A), prompting us to explore MeltArray’s potential for quantifying the MUT% by leveraging the Rm values. Using D94G as a model, we plotted Rm values against MUT% ranging from 1% to 100% across five DNA concentrations (5 × 10¹ to 5 × 10⁵ copies/μL), achieving an exponential relationship (R² of 0.99, Fig. S7B). By limiting MUT% in certain ranges (10%–90%, 10%–80%, 10%–70%, 20%–90%, 20%–80%, and 20%–70%), a linear relationship between Rm and MUT% could be obtained without significant differences in model performance (*P* > 0.05; Table S8). To perform quantification over the broadest range, the linear regression curve in the 10%–90% range was selected for MUT% calculation (Fig. S7B).

**Fig 5:**
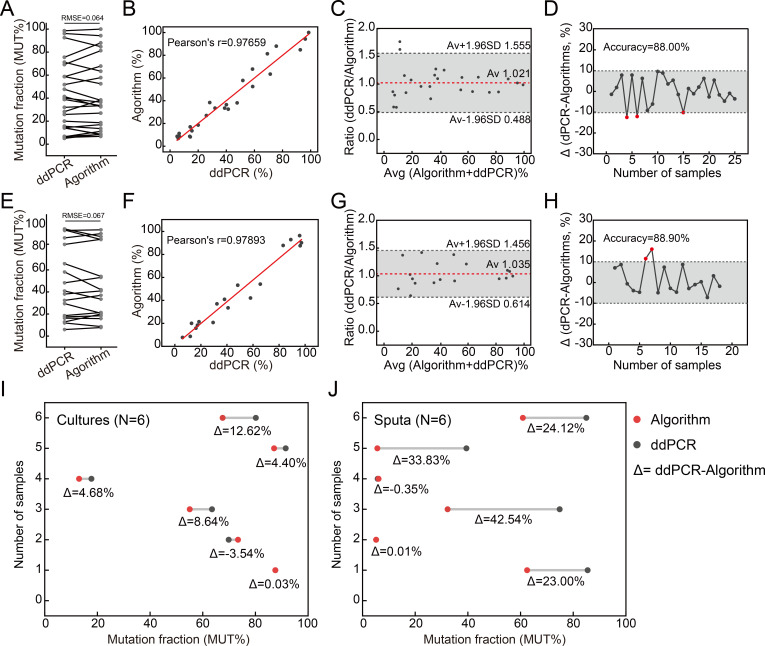
Prediction of mutation fractions of D94G based on the polynomial regression algorithm-based formula. RMSE (**A**), Pearson correlation analysis (**B**), Bland-Altman analysis (**C**), and best prediction accuracy (**D**) in the training set. RMSE (**E**), Pearson correlation analysis (**F**), Bland-Altman analysis (**G**), and best prediction accuracy (**H**) in the validation set. Twelve D94G heterogeneous samples from Group 2 and Group 3 were selected as a test set to evaluate the algorithm’s accuracy, comprising six culture samples (**I**) and six sputum samples (**J**).

Using ddPCR results as the reference, the algorithm achieved 88.00% accuracy in the training set, with an optimal *k* of 0.49 and a RMSE of 0.065, demonstrating minimal prediction error and robust predictive capability (Fig. 5A and D). Pearson correlation (r = 0.97659) and Bland-Altman analysis (mean ratio = 1.021) confirmed its strong correlation and consistency with ddPCR in quantifying MUT% (Fig. 5B and C). Validation on 18 additional samples further supported robust performance (Fig. 5E through G), with accurate predictions in 88.90% of heteroresistant mutations (Fig. 5H). Analysis of 12 D94G heterogeneous mutations from Group 2 and Group 3 revealed high accuracy in culture samples (5/6, 83.33%), one culture sample showed a deviation of 12.62%, underscoring the algorithm’s reliability (Fig. 5I). However, a marked discrepancy was observed in sputum samples (2/6, 33.33%), particularly in samples with higher MUT%, where the algorithm’s predictions were 20% to 45% lower than ddPCR measurements (Fig. 5J).

## DISCUSSION

We have developed the MeltArray MTB/FQs assay, a novel molecular tool capable of identifying 11 key *gyrA*-QRDR resistance mutations in a single reaction. The assay employs “Fluorescence-*T_m_*” codes that not only can determine individual mutation types but also resolve multiple co-occurring mutations within a sample. Such an advantage, coupled with a LOD of 50 copies/reaction, ensures MeltArray’s sensitivity in detecting heteroresistant mutation as low as 5% across a broader concentration range (50-50,000 copies/reaction). Clinical evaluations showed that MeltArray outperformed Sanger sequencing and the commercial MeltPro MTB/FQs kit in sensitivity and accuracy for both culture and sputum samples. Its streamlined one-step reaction offers a significant advantage over time-consuming and resource-intensive methods like next-generation sequencing (NGS) (25). Within 3 h, the assay provides both qualitative and quantitative mutation data, making it an efficient alternative for routine FQs resistance monitoring, where timely decision-making can significantly impact patient outcomes.

A key strength of MeltArray lies in its ability to accurately detect multi-mutant samples. This assay produces distinct “Fluorescence-*T_m_*” codes for each mutation, enabling the resolution of overlapping *T_m_* peaks that can confound by traditional approaches (23). This is a significant improvement over earlier assays, which relied on dual *T_m_* codes generated by sloppy molecular beacons (12, 17). While these methods were effective in detecting single mutations, they struggled with multi-mutant profiles due to difficulties in deconvoluting overlapping *T_m_* peaks, leading to inaccurate or ambiguous results (12, 17, 19).

MeltArray also excels in detecting low-fraction heteroresistance, a common challenge in detecting FQs-resistant mutations (12, 17, 31, 32). In our study, up to 19.93% (56/281) of mutant MTB isolates exhibited heteroresistance, a phenomenon often missed by conventional diagnostic methods that require mutations to be present at higher fractions for detection (12, 17, 22). In the clinical evaluation of Group 1 samples, which included 56 multi-mutant samples, MeltArray identified 98.57% (138/140) of mutations, with 42.03% (58/138) of these mutations present at fractions below 20%. In comparison, Sanger sequencing identified 71.43% (100/140) of these mutations. The ability to detect low-fraction heteroresistance renders the superior sensitivity of MeltArray in identifying resistant strains, allowing for revealing FQs resistance in patients at early stages.

Direct detection of sputum samples represents a promising approach for rapid FQs resistance screening. However, challenges, such as low bacterial loads and PCR inhibitors often complicate testing (33, 34). To address these problems, we incorporated a Cq value as a quality control parameter to ensure the reliability of the assay in sputum samples. As shown in Group 2, MeltArray detected all mutations above the LOD-HR in FQs-resistant sputum samples with Cq ≤ 35, while only one mutation was detected in those with Cq > 35. This observation suggests that the Cq cutoff enhances the reliability of MeltArray for sputum testing, ensuring that only high-quality samples are analyzed. Furthermore, as evidenced by ddPCR, MeltArray showed slightly better accuracy when compared with the commercial MeltPro MTB/FQs kit in Group 3, further validating its potential as a rapid alternative for FQs resistance screening.

Despite these strengths, there are several limitations to the MeltArray assay that warrant consideration. First, while the assay exhibited robust performance in culture specimens, its success rate was reduced in sputum specimens, where low MTB load and complex matrix composition led to a 20%–30% failure rate in quality control (Cq > 35). This may result in delayed reporting of results for these cases. To enhance clinical applicability for sputum specimens with low MTB loads, further optimizations will be needed in pre-analytical procedures, including the gDNA extraction protocol and pre-enrichment of extracted gDNA, as well as in the analytical phase, including gDNA input volume and reaction components. Second, while the MeltArray assay is effective for detecting mutations present at levels greater than 5%, it may miss heteroresistance below this threshold, particularly when compared with more sensitive techniques like NGS and ddPCR, which can detect mutations at fractions lower than 1%. However, only 2.85% (8/281) of the specimens in our study exhibited heteroresistance in the 1%-5% range, suggesting that the LOD-HR of 5% is sufficient for most clinical applications. Third, the algorithm developed to quantify MUT% also shows promise, though some bias remains when compared with ddPCR. Future research will focus on refining the algorithm to improve its reliability. Last, while the MeltArray assay has shown excellent performance in detecting *gyrA*-QRDR mutations, additional research is needed to determine whether it can be extended to other clinically relevant mutations and broader drug resistance profiles.

In conclusion, the MeltArray MTB/FQs assay offers a rapid, highly sensitive, and accurate method for detecting FQs resistance of MTB. Its ability to detect multi-mutant strains, identify low-level heteroresistance, and quantify mutation fractions makes it an invaluable tool for clinical diagnostics. With its streamlined protocol, quick turnaround time, and exceptional sensitivity, MeltArray stands out as a promising alternative for routine FQs resistance monitoring, with the potential to improve clinical decision-making and patient outcomes in the management of pre-XDR-TB.

## SUPPLEMENTAL MATERIAL

File S1 to S3, Tables S1 to S8, and Fig. S1 to S7 jcm.00146-25-s0001.pdf File S1: Bactec MGIT960 Liquid Culture. File S2: MTB Real-Time PCR Test. File S3: MeltPro MTB/FQs Assay. Table S1: Primers and probes used in the MeltArray MTB/FQs. Table S2: Information of 24 NTM and 18 non-mycobacterial. Table S3: *T*_*m*_ reproducibility of MeltArray MTB/FQs and interpretive threshold values for 11 mutations. Table S4: Discordance between pAST and MeltArray MTB/FQs in 442 culture samples from Group 1. Table S5: Comparison of the mutation identification results between the MeltArray MTB/FQs and Sanger sequencing in 56 multi-mutant samples from Group 1. Table S6: Discordant samples between MeltArray MTB/FQs and Sanger sequencing were verified using ddPCR in 56 multi-mutant samples from Group 1. Table S7: Discordant results between MeltPro and MeltArray in sputum samples from Group 3. Table S8: R² Values of the fitted relationship curves between Rm and MUT% across different regions. Fig. S1: Evaluation of the analytical performance of MeltArray MTB/FQs. Fig. S2: Evaluation of the limit of detection (LOD) for the MeltArray MTB/FQs assay using intact *Mycobacterium tuberculosis* (MTB) strains in culture and sputum specimens. Fig. S3: A 2×2 contingency table comparing the performance of Sanger sequencing with pAST in 442 culture samples from Group 1. Fig. S4: 2×2 contingency tables comparing the performance of Sanger sequencing with pAST in 121 paired sputum-culture samples from Group 2. Fig. S5: Comparison of the identification results between the MeltArray MTB/FQs and Sanger sequencing in 121 paired sputum-culture samples. Fig. S6: Distribution of the Cq value of sputum samples among different positive values obtained by microscopic examination and liquid culture samples. Fig. S7: MeltArray analysis was conducted at five different genomic concentrations, using *gyrA* D94G as an example.

ASM does not own the copyrights to Supplemental Material that may be linked to, or accessed through, an article. The authors have granted ASM a non-exclusive, world-wide license to publish the Supplemental Material files. Please contact the corresponding author directly for reuse.
