# Supplementary material for: Identification and quantification of gyrA variants in fluoroquinolone-resistant Mycobacterium tuberculosis in a MeltArray reaction
Source: J Clin Microbiol. 2025 Jun 3;63(7):e00146-25. doi: 10.1128/jcm.00146-25 (PMC12239727; doi:10.1128/jcm.00146-25)
Supplement: File S1 to S3, Tables S1 to S8, and Fig. S1 to S7 — File S1: Bactec MGIT960 Liquid Culture. File S2: MTB Real-Time PCR Test. File S3: MeltPro MTB/FQs Assay. Table S1: Primers and probes used in the MeltArray MTB/FQs. Table S2: Information of 24 NTM and 18 non-mycobacterial. Table S3: Tm reproducibility of MeltArray MTB/FQs and interpretive threshold values for 11 mutations. Table S4: Discordance between pAST and MeltArray MTB/FQs in 442 culture samples from Group 1. Table S5: Comparison of the mutation identification results between the MeltArray MTB/FQs and Sanger sequencing in 56 multi-mutant samples from Group 1. Table S6: Discordant samples between MeltArray MTB/FQs and Sanger sequencing were verified using ddPCR in 56 multi-mutant samples from Group 1. Table S7: Discordant results between MeltPro and MeltArray in sputum samples from Group 3. Table S8: R² Values of the fitted relationship curves between Rm and MUT% across different regions. Fig. S1: Evaluation of the analytical performance of MeltArray MTB/FQs. Fig. S2: Evaluation of the limit of detection (LOD) for the MeltArray MTB/FQs assay using intact Mycobacterium tuberculosis (MTB) strains in culture and sputum specimens. Fig. S3: A 2×2 contingency table comparing the performance of Sanger sequencing with pAST in 442 culture samples from Group 1. Fig. S4: 2×2 contingency tables comparing the performance of Sanger sequencing with pAST in 121 paired sputum-culture samples from Group 2. Fig. S5: Comparison of the identification results between the MeltArray MTB/FQs and Sanger sequencing in 121 paired sputum-culture samples. Fig. S6: Distribution of the Cq value of sputum samples among different positive values obtained by microscopic examination and liquid culture samples. Fig. S7: MeltArray analysis was conducted at five different genomic concentrations, using gyrA D94G as an example. [file jcm.00146-25-s0001.pdf]

Supplemental Materials for:

Identification and Quantification of *gyrA* Variants in Fluoroquinolone-Resistant *Mycobacterium tuberculosis* in a MeltArray Reaction

File S1. Bactec MGIT960 Liquid Culture

To identify MTB from culture-positive samples, we followed the Chinese National Laboratory Guidelines for Tuberculosis Diagnosis and adhered to the standard operating procedures of the MGIT 960 system (Becton, Dickinson, MD, USA). Specifically, culture-positive samples were initially examined using Ziehl-Neelsen staining. If acid-fast bacilli were observed, the culture was considered positive for mycobacteria. Species identification was subsequently performed using the MPB64 colloidal gold assay. Briefly, 20 μL of bacterial suspension was mixed with 0.5 mL of saline, vortexed, and 100 μL of the mixture was applied to the test strip. Results were interpreted as follows: a positive result was indicated by purple-red bands in both the test and control zones; a negative result showed a purple-red band only in the control zone; absence of a control band rendered the test invalid and required retesting. For MPB64-negative isolates, further differentiation was conducted by inoculating 100 μL of bacterial suspension onto modified Löwenstein-Jensen (L-J) and para-nitrobenzoic acid (PNB) media. Growth on both media indicated NTM, whereas growth only on L-J medium confirmed the presence of *Mycobacterium tuberculosis* complex (MTBC).

File S2. MTB Real-Time PCR Test

The MTB real-time PCR test was used to identify MTB in all sputum samples, which purchased from Zeesan Biotechnology Co., Ltd. (Xiamen, China). Following the instructions, 30 μL template was added to PCR tube, sealed, vortexed for 20 seconds, and then placed in the SLAN-96 thermocycler for amplification. The amplification program started with a contamination control procedure of 2 min at 50 °C to prevent carry-over of DNA amplicon using uracil-N-glycosylase, followed by a denaturation at 95 °C for 10 min, 10 cycles 95 °C for 10 s, 68 °C for 15 s (-1 °C/cycle) and 72 °C for 15 s, and 45 cycles of 95 °C for 10 s, 58 °C for 20 s and 72 °C for 15 s. Fluorescence was recorded in both the FAM and HEX channels, with results were interpreted automatically by the SLAN software version 8.2.

File S3. MeltPro MTB/FQs Assay

The MeltPro MTB/FQs kit, purchased from Zeesan Biotechnology Co., Ltd (Xiamen, China), was used to detect FQs resistance of sputum samples in Group 3. This kit, based on multi-color melting curve analysis, employs two TaqMan probes designed to cross-cover codons 88 to 94 within the quinolone resistance-determining region (QRDR) of the *gyrA* gene, enabling the identification of MTB resistance to FQs.

Detections were conducted according to the manufacturer's instructions. Briefly, 5 μL gDNA template was added to the 20 μL prepared mixtures. The PCR was performed on the SLAN-96S thermocycler following the application protocols: a contamination control step for 2 min at 50 °C to prevent carry-over of DNA amplicon using uracil-N-glycosylase, followed by a denaturation at 95 °C for 10 min, 10 cycles 95 °C for 10 s, 65 °C for 20 s (-1 °C/cycle) and 78 °C for 25 s, and 40 cycles of 95 °C for 10 s, 55 °C for 20 s and 78 °C for 25 s. After PCR amplification, a melting curve analysis was initiated with 2 min of denaturation at 95°C, 2 min of hybridization at 40°C, and a stepwise increasing temperature from 40°C to 85°C with collection of the fluorescent signal intensity for the FAM channel, with results were interpreted automatically by the SLAN software version 8.2 (Hongshi, Shanghai, China).

Table S1 Primers and probes used in the MeltArray MTB/FQs.

Oligo name	Sequence(5' to 3')[a]	Concentration(pmol)
gyrB-F	ACTCGGACGCGTATGCG	10
gyrB-R	CGAGCCGGGTGGATAGC	10
gyrA-F	TATGCAATGTTCGATTCCGG	10
gyrA-R	CTGGCGAGCCGAAGT	10
gyrB-LP	HEX-TGGTGGTCTGCACGGCGTCGGCGT-BHQ1	0.5
G88A-MP	**GTGCTCACCTG**CGTGCGGGTGGTAGTTGCCCAT	1
G88C-MP	**TGGTCTATCTTTTA**GTGCGGGTGGTAGTTGCCCATGGTCC	10
D89N-MP	**TCACACTGATCTGT**GSMGTGCGGGTGGTAGTTGCC	4
D89G-MP	**ACCTCTCACAC**CGSMGTGCGGGTGGTAGTTGCC	1
A90V-MP	**CTCTCTGGTCTAT**GKCGATCTACNVCASCCTGGTGCGC	10
S91P-MP	**CCTGCCGTGC**CGATCTACNVCASCCTGGTGC	8
D94G-MP	**CACTGTCCAG**GCASCCTGGTGCGCAT	20
D94A-MP	**AGCCGTGTCC**GATCTACGCCASCCTGGT	10
D94N-MP	**ATCACACCTC**AACASCCTGGTGCGC	4
D94H-MP	**AC*G*ACGGTCC**TACCACASCCTGGTGCGCAGGGTCGT	4
D94Y-MP	**CCTCCTGCCA**CTACASCCTGGTGCGC	4

[a]Bold nucleotides indicate the mediator primer sequences; * indicates thiophosphate bonds.

Table S2 Information of 24 NTM and 18 non-mycobacterial.

No.	NTM	No.	Non-mycobacterial
1	*Mycobacterium kansasii*	1	*Streptococcus pneumoniae*
2	*Mycobacterium marinum*	2	*Bordetella pertussis*
3	*Mycobacterium simiae*	3	*Klebsiella pneumoniae*
4	*Mycobacterium lentiflavum*	4	*Escherichia coli*
5	*Mycobacterium asiaticum*	5	*Pseudomonas aeruginosa*
6	*Mycobacterium scrofulaceum*	6	*Acinetobacter baumannii*
7	*Mycobacterium gordonae*	7	*Staphylococcus aureus*
8	*Mycobacterium szulgai*	8	*Enterobacter cloacae*
9	*Mycobacterium avium*	9	Adenovirus
10	*Mycobacterium intracellulare*	10	*Citrobacter freundii*
11	*Mycobacterium xenopi*	11	*Mycoplasma pneumoniae*
12	*Mycobacterium ulcerans*	12	*Morganella morganii*
13	*Mycobacterium terrae*	13	*Klebsiella oxytoca*
14	*Mycobacterium nonchromogenicum*	14	*Streptococcus anginosus*
15	*Mycobacterium shimoidei*	15	*Enterococcus faecalis*
16	*Mycobacterium malmoense*	16	*Serratia marcescens*
17	*Mycobacterium farcinogenes*	17	*Crytococcus neofonmans*
18	*Mycobacterium fortuitum*	18	*Aeromonas hydrophila*
19	*Mycobacterium phlei*		
20	*Mycobacterium aichiense*		
21	*Mycobacterium aurum*		
22	*Mycobacterium chubuense*		
23	*Mycobacterium duvalii*		
24	*Mycobacterium flavescens*		

Table S3 T_m reproducibility of MeltArray MTB/FQs and interpretive threshold values for 11 mutations.

Channel	Mutation type	Codon change	T_m (°C) (Mean ± 3SD)	CV (%)	Threshold value
FAM	G88A	GGC > GCC	78.77 ± 0.29	0.12	0.19
HEX	G88C	GGC > TGC	59.38 ± 0.30	0.17	0.18
FAM	D89N	GAC > AAC	58.19 ± 0.27	0.16	0.31
ROX	D89G	GAC > GGC	64.62 ± 0.33	0.17	0.30
HEX	A90V	GCG > GTG	64.69 ± 0.36	0.18	0.27
FAM	S91P	TCG > CCG	82.27 ± 0.48	0.19	0.38
ROX	D94G	GAC > GGC	81.23 ± 0.23	0.09	0.16
HEX	D94A	GAC > GCC	78.35 ± 0.41	0.18	0.18
Cy5	D94Y	GAC > TAC	80.39 ± 0.18	0.08	0.29
Cy5	D94H	GAC > CAC	73.45 ± 0.25	0.11	0.31
ROX	D94N	GAC > AAC	69.55 ± 0.41	0.20	0.36

Table S4 Discordance between pAST and MeltArray MTB/FQs in 442 culture samples from Group 1.

Sample ID	pAST	MeltArray MTB/FQs	Sanger sequencing of *gyrA*-QRDR	Sanger sequencing of *gyrB* (codons 461 to 499)
**pAST-R vs MeltArray-WT（N=14）**				
2-4	R	*gyrA*-WT	*gyrA*-WT	*gyrB*-WT
2-35	R	*gyrA*-WT	*gyrA*-WT	*gyrB*-WT
4-16	R	*gyrA*-WT	*gyrA*-WT	*gyrB*-WT
4-23	R	*gyrA*-WT	*gyrA*-WT	*gyrB*-WT
4-29	R	*gyrA*-WT	*gyrA*-WT	*gyrB*-WT
4-37	R	*gyrA*-WT	*gyrA*-WT	*gyrB*-WT
4-38	R	*gyrA*-WT	*gyrA*-WT	*gyrB*-WT
4-57	R	*gyrA*-WT	*gyrA*-WT	*gyrB*-D461N
4-61	R	*gyrA*-WT	*gyrA*-WT	*gyrB*-WT
4-65	R	*gyrA*-WT	*gyrA*-WT	*gyrB*-WT
4-72	R	*gyrA*-WT	*gyrA*-WT	*gyrB*-WT
4-86	R	*gyrA*-WT	*gyrA*-WT	*gyrB*-WT
4-90	R	*gyrA*-WT	*gyrA*-WT	*gyrB*-WT
4-120	R	*gyrA*-WT	*gyrA*-WT	*gyrB*-D461N
**pAST-S vs MeltArray-MUT（N=1）**				
94095	S	*gyrA*-D94G	*gyrA*-D94G	*gyrB*-WT

Note: MUT indicates mutant type, WT indicates wild type.

Table S5 Comparison of the mutation identification results between the MeltArray MTB/FQs and Sanger sequencing in 56 multi-mutant samples from Group 1.

No.	MeltArray MTB/FQs	Sanger sequencing	Consistency	No.	MeltArray MTB/FQs	Sanger sequencing	Consistency
1	A90V+D94A	A90V+D94A	Yes	29	D94A+A90V+D94G	D94G+D94A	No
2	D94A+A90V+D94G	D94A+A90V+D94G	Yes	30	D94G+D94N	D94N+D94G	Yes
3	D94G+D94A	D94G+D94A	Yes	31	A90V+D94N+S91P	A90V+D94N+S91P	Yes
4	A90V+D94G	A90V+D94G	Yes	32	D94G+A90V+D94A+D89G+S91P	A90V+D94G+D94A	No
5	D94A+A90V+D94G	D94A+A90V+D94G	Yes	33	D94G+D94A+D94Y	D94G+D94Y+D94A	Yes
6	A90V+D94G	A90V+D94G	Yes	34	A90V+S91P+D94G	A90V	No
7	A90V+D94A	A90V+D94A	Yes	35	A90V+G88A	A90V+G88A	Yes
8	D94A+S91P	D94A	No	36	A90V+D94A	D94A	No
9	D94G+D94A	D94G	No	37	A90V+S91P+D94H	A90V+D94H+S91P	Yes
10	D94G+D94A	D94A	No	38	D94A+A90V+D94G	A90V+D94G+D94A	Yes
11	D94G+D94A	D94G	No	39	A90V+D94N	A90V+D94N	Yes
12	G88C+A90V+D94A+D94N+D94G	G88C+A90V+D94N+D94G	No	40	D94G+S91P	D94G+S91P	Yes
13	A90V+D94A	D94A	No	41	D94G+A90V+S91P+D94N	D94G+D94N	No
14	D94N+D94H	D94N	No	42	A90V+D94H	A90V+D94H	Yes
15	A90V+D94G	D94G	No	43	D94A+A90V+D94G	D94A+A90V+D94G	Yes
16	A90V+D94G	A90V+D94G	Yes	44	A90V+D94G	A90V+D94G	Yes
17	D94G+D94A	D94A	No	45	A90V+D94G	A90V+D94G	Yes
18	D94G+D94A	D94A	No	46	A90V+D94G	A90V	No
19	A90V+D94G	D94G	No	47	A90V+D94G	D94G	No
20	D94A+S91P	D94A+S91P	Yes	48	A90V+D94A+S91P+D94G	A90V+D94A+D94G	No
21	A90V+D94G	D94G	No	49	D94G+A90V+S91P+D94N	D94G+D94N	No
22	D94A+D89N	D94A+D89N	Yes	50	D94Y+D94A	D94Y	No
23	A90V+D94A	A90V+D94A	Yes	51	D94G+S91P	D94G+S91P	Yes
24	A90V+S91P	A90V+S91P	Yes	52	A90V+D94A+S91P+D94G	A90V	No
25	D94G+D94A	WT	No	53	A90V+D94Y	A90V+D94Y	Yes
26	A90V+S91P	S91P	No	54	D94G+D94A	WT	No
27	D94A+A90V+D94G	D94A	No	55	D94A+A90V+D94G	D94A+A90V+D94G	Yes
28	D94A+A90V+D94G	D94A+D94G	No	56	A90V+D94G	WT	No

Note: "Yes" indicates that the mutation identification result between the MeltArray MTB/FQs and Sanger sequencing is consistency, "No" indicates that the mutation identification result between the MeltArray MTB/FQs and Sanger sequencing is inconsistency.

Table S6 Discordant samples between MeltArray MTB/FQs and Sanger sequencing were verified using ddPCR in 56 multi-mutant samples from Group 1.

No.	Sanger sequencing	MeltArray MTB/FQs	ddPCR (Heteroresistance)	Consistency between MeltArray and ddPCR
1	D94A	D94A+S91P	D94A(92.10%)+S91P(7.00%)	Yes
2	D94G	D94G+D94A	D94G(89.00%)+D94A(10.60%)	Yes
3	D94A	D94G+D94A	**A90V(0.92%)**+D94A(87.43%)+D94G(12.83%)	No
4	D94G	D94G+D94A	**A90V(1.12%)**+D94A(1.73%)+D94G(97.15%)	No
5	G88C+A90V+D94N+D94G	G88C+A90V+D94A+D94N+D94G	D94G(18.53%)+D94A&D94N(33.36%)+A90V&G88C(46.35%)	Yes
6	D94A	A90V+D94A	A90V(4.74%)+D94A(95.18%)	Yes
7	D94N	D94N+D94H	D94N(84.87%)+D94H(15.21%)	Yes
8	D94G	A90V+D94G	D94G(92.60%)+A90V(6.90%)	Yes
9	D94A	D94G+D94A	D94A(54.99%)+D94G(14.42%)	Yes
10	D94A	D94G+D94A	D94A(69.08%)+D94G(14.36%)	Yes
11	D94G	A90V+D94G	D94G(75.54%)+A90V(22.36%)	Yes
12	D94G	A90V+D94G	A90V(42.78%)+D94G(58.77%)	Yes
13	WT	D94G+D94A	D94A(7.14%)+D94G(5.85%)	Yes
14	S91P	A90V+S91P	S91P(94.86%)+A90V(5.11%)	Yes
15	D94A	D94A+A90V+D94G	A90V(3.40%)+D94A(79.19%)+D94G(6.73%)	Yes
16	D94A+D94G	D94A+A90V+D94G	D94A(52.80%)+D94G(32.32%)+A90V(10.30%)	Yes
17	D94G+D94A	D94A+A90V+D94G	A90V(8.96%)+D94A(20.08%)+D94G(40.08%)	Yes
18	A90V+D94A+D94G+D89G	D94G+A90V+D94A+D89G+S91P	D94G(38.11%)+D94A(11.34%)+A90V(10.30%)+D89G(20.00%)+S91P(3.41%)	Yes
19	A90V	A90V+S91P+D94G	D94G(11.85%)+A90V(79.78%)+S91P(6.83%)	Yes
20	D94A	A90V+D94A	D94A(89.52%)+A90V(10.28%)	Yes
21	D94N+D94G	D94G+A90V+S91P+D94N	A90V(19.04%)+S91P(2.77%)+D94G(58.09%)+D94N(18.57%)	Yes
22	A90V	A90V+D94G	A90V(79.39%)+D94G(17.39%)	Yes
23	D94G	A90V+D94G	D94G(96.20%)+A90V(3.57%)	Yes
24	A90V+D94A+D94G	A90V+D94A+S91P+D94G	A90V(17.40%)+D94G(19.95%)+D94A(50.30%)+S91P(8.74%)	Yes
25	D94G+D94N	D94G+A90V+S91P+D94N	S91P(3.43%)+A90V(8.13%)+D94N(14.73%)+D94G(71.26%)	Yes
26	D94Y	D94Y+D94A	D94Y(94.41%)+D94A(5.43%)	Yes
27	A90V	A90V+D94A+S91P+D94G	S91P(23.29%)+A90V(54.39%)+D94G(14.04%)+D94A(6.08%)	Yes
28	WT	D94G+D94A	D94G(6.50%)+D94A(8.10%)	Yes
29	WT	A90V+D94G	A90V(4.47%)+D94G(5.90%)	Yes

Note: "Yes" indicates that the mutation identification result between the MeltArray MTB/FQs and ddPCR is consistency, "No" indicates that the mutation identification result between the MeltArray MTB/FQs and ddPCR is inconsistency.

Table S7 Discordant results between MeltPro and MeltArray in sputum samples from Group 3.

Sample ID	Cq	MeltArray MTB/FQs	MeltPro MTB/FQs	ddPCR (Heteroresistance)
**MeltArray-MUT vs MeltPro-WT (N=4)**				
2-13	31.93	D94A	WT	D94A (100%)
2-22	31.41	D94G	WT	D94G (80.97%)
2-458	27.54	D94N	WT	D94N (10.31%)
2-1787	20.30	D94G	WT	D94G (100%)
**MeltArray-WT vs MeltPro-MUT (N=3)**				
2-8	34.15	WT	MUT	D94G (100%)
2-106	34.12	WT	MUT	WT (100%)
2-457	34.14	WT	MUT	WT (100%)

Note: MUT indicates mutant type, WT indicates wild type.

Table S8 R^2 Values of the fitted relationship curves between Rm and MUT% across different regions.

**Region**	**5×10^5 copies/μL**	**5×10^4 copies/μL**	**5×10^3 copies/μL**	**5×10^2 copies/μL**	**5×10^1 copies/μL**	**Average**	***P* value**
[10%, 90%]	0.99332	0.99728	0.99542	0.99492	0.99876	0.99594	……
[10%, 80%]	0.99253	0.99871	0.99585	0.99628	0.99871	0.99642	0.741
[10%, 70%]	0.98609	0.99769	0.99778	0.99663	0.99846	0.99515	0.852
[20%, 90%]	0.99420	0.99656	0.97900	0.99080	0.99754	0.99162	0.701
[20%, 80%]	0.99362	0.99933	0.98193	0.99291	0.99724	0.99301	0.481
[20%, 70%]	0.98923	0.99905	0.99889	0.99922	0.99657	0.99659	0.264

Note: p value < 0.05 considered statistically significant.

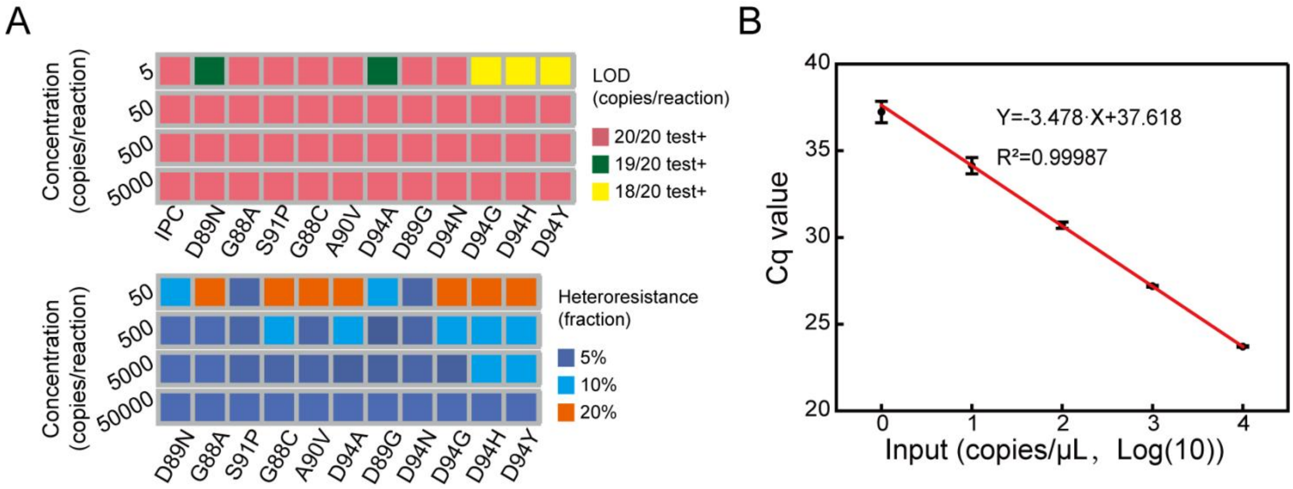

Fig S1 Evaluation of the analytical performance of MeltArray MTB/FQs. (A) The repeatability test for LOD calculation (N = 20) was performed for the IPC and 11 *gyrA*-QRDR mutations. The LOD-HR for these 11 mutations was detected across four concentrations, ranging from 50 to 50,000 copies/reaction. (B) Distribution of Cq values across five different concentrations.

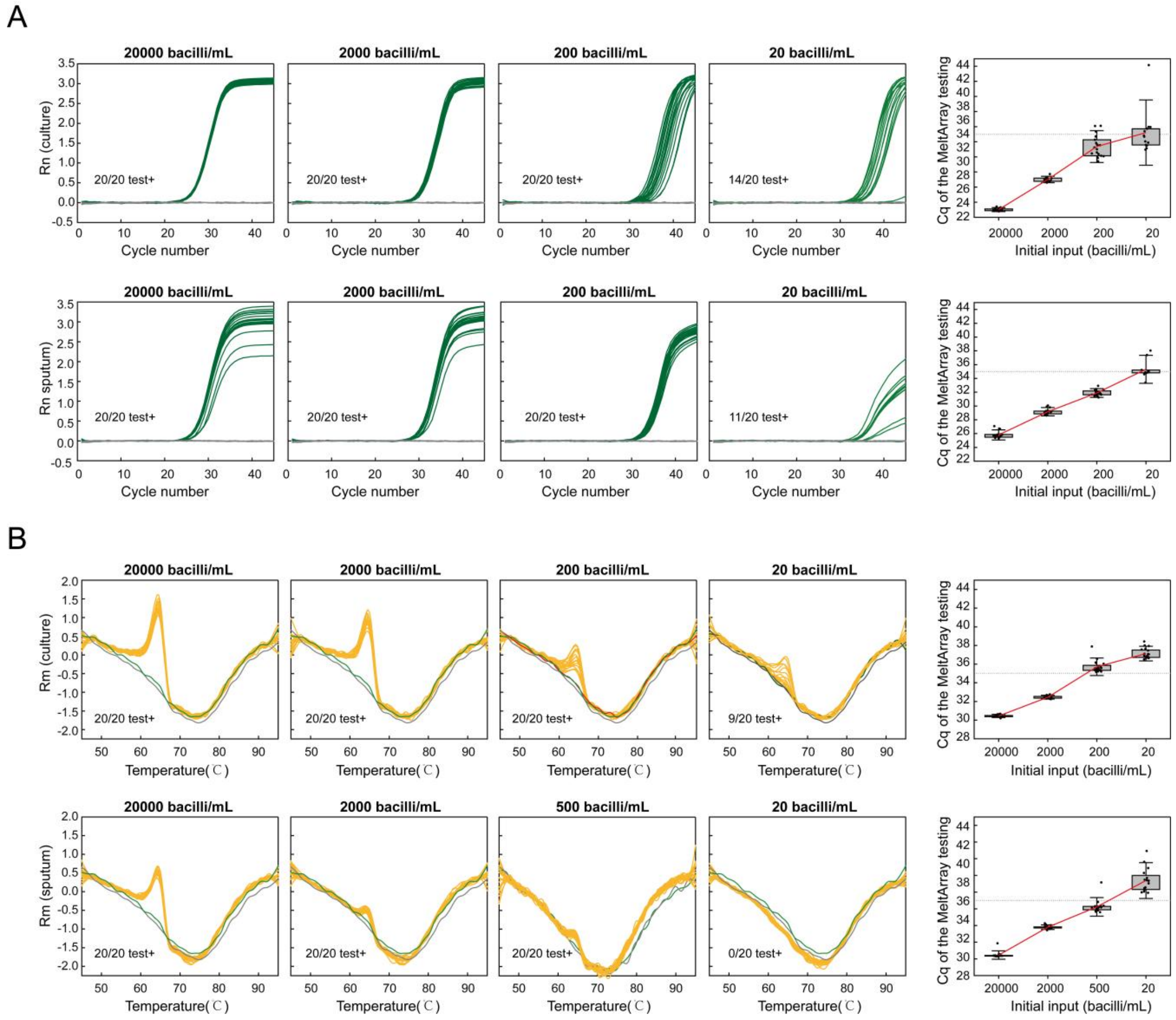

Fig S2 Evaluation of the limit of detection (LOD) for the MeltArray MTB/FQs assay using intact *Mycobacterium tuberculosis* (MTB) strains in culture and sputum specimens. (A) Real-time PCR amplification curves of the wild-type MTB strain (H37Rv) spiked into culture (top row) and MTB-negative sputum specimens (bottom row) at concentrations of 20,000, 2,000, 200, and 20 bacilli/mL. Amplification curves for each concentration are shown as green lines, while the negative control (no template control, NTC) is represented by gray lines. In our study, the MeltArray strategy employed two-dimensional markers, Fluorescence-T_m, to identify mutations based on distinct melting peaks, with wild-type strains showing no melting peaks. Although the figure does not display melting curves, the actual results show that no melting peaks were observed across all four fluorescence channels. The corresponding boxplots (right panels) show the distribution of Cq values at each bacterial input level. (B) Melting curve profiles of the *gyrA* A90V mutant strain in culture (top row) and sputum specimens (bottom row). While sputum testing was initially performed at the same concentrations (20,000 to 20 bacilli/mL), no discernible melting peaks were observed at 200 bacilli/mL. An intermediate concentration of 500 bacilli/mL was therefore included, at which melting peaks became clearly detectable. Melting curves for each concentration are shown as yellow lines, while the wild-type is represented by green line and NTC is represented by gray line. The corresponding boxplots (right panels) show the distribution of Cq values at each bacterial input level. In each boxplot, boxes represent the mean ± 2 standard deviations (SD). The red solid line connects the means, while the grey dotted line denotes the Cq cut-off threshold (Cq = 35).

Sanger sequencing	pAST: R	pAST: S
MUT	275	1
WT	19	147

Sensitivity: 93.54%
Specificity: 99.32%

Fig S3 A 2×2 contingency table comparing the performance of Sanger sequencing with pAST in 442 culture samples from Group 1.

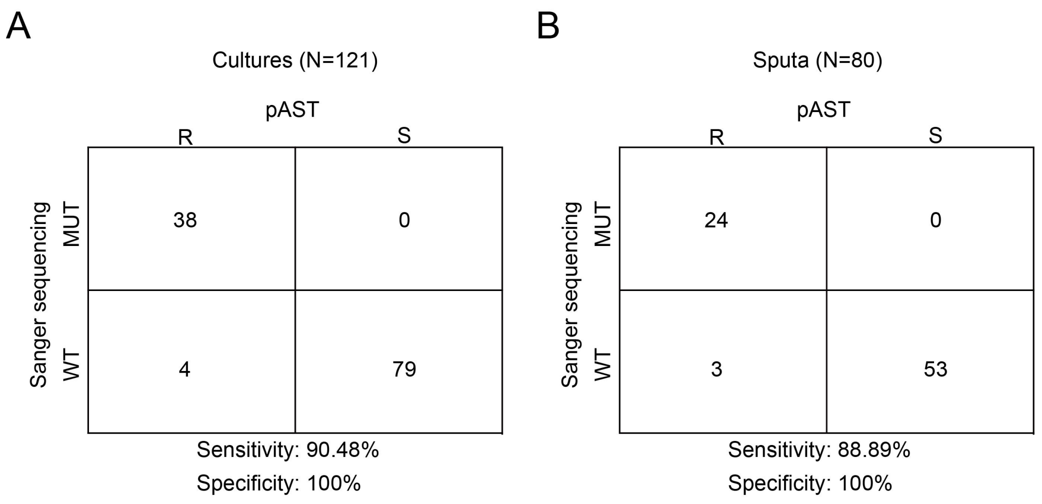

Fig S4 2×2 contingency tables comparing the performance of Sanger sequencing with pAST in 121 paired sputum-culture samples from Group 2. (A) Comparison of Sanger sequencing results in 121 quality-controlled culture samples (Cq < 35) against pAST. (B) Comparison of Sanger sequencing results in 80 quality-controlled sputum samples (Cq < 35) against pAST.

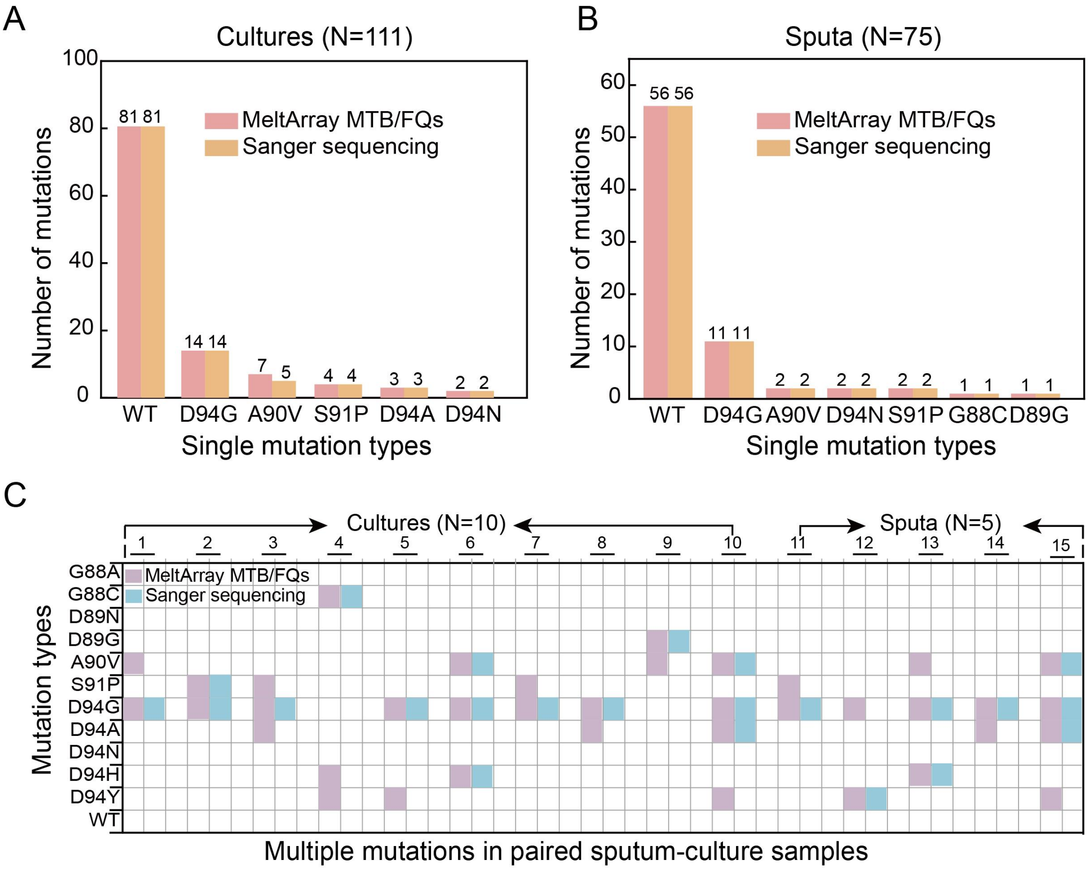

Fig S5 Comparison of the identification results between the MeltArray MTB/FQs and Sanger sequencing in 121 paired sputum-culture samples. (A) Comparison of identification results between MeltArray assay and Sanger sequencing in culture samples classified as wild-type or single mutations by MeltArray (N = 111). (B) Comparison of identification results between MeltArray assay and Sanger sequencing in sputum samples classified as wild-type or single mutations by MeltArray (N = 75). (C) Comparison of identification results between MeltArray assay and Sanger sequencing in 15 multi-mutation samples, including 10 culture samples and 5 sputum samples.

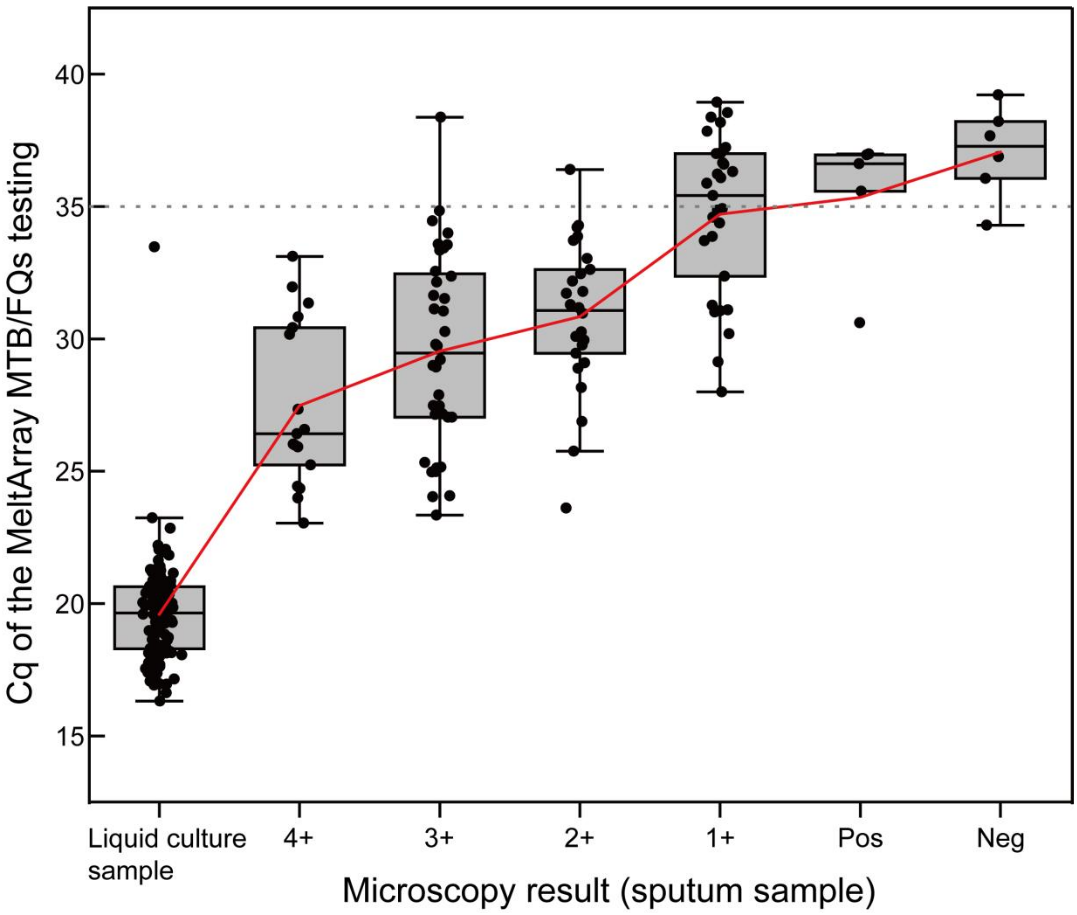

Fig S6 Distribution of the Cq value of sputum samples among different positive values obtained by microscopic examination and liquid culture samples. Smear-positive (4+, 3+, 2+, 1+) refers to specimens classified according to standard smear grading, whereas smear-positive (Pos) indicates samples with only a few bacilli observed on smear microscopy, insufficient to qualify for the grading. Smear-negative (Neg) refers to samples in which no bacilli were observed under microscopy. The cut-off Cq value is 35 (gray dotted line). For box plots, centerlines indicate medians, box limits represent the first and third quartiles (Q1 and Q3), and whiskers extend to the most extreme data points within 1.5 times the interquartile range (IQR). The red solid line connects the mean Cq values for each group, illustrating the overall trend across different smear grading categories.

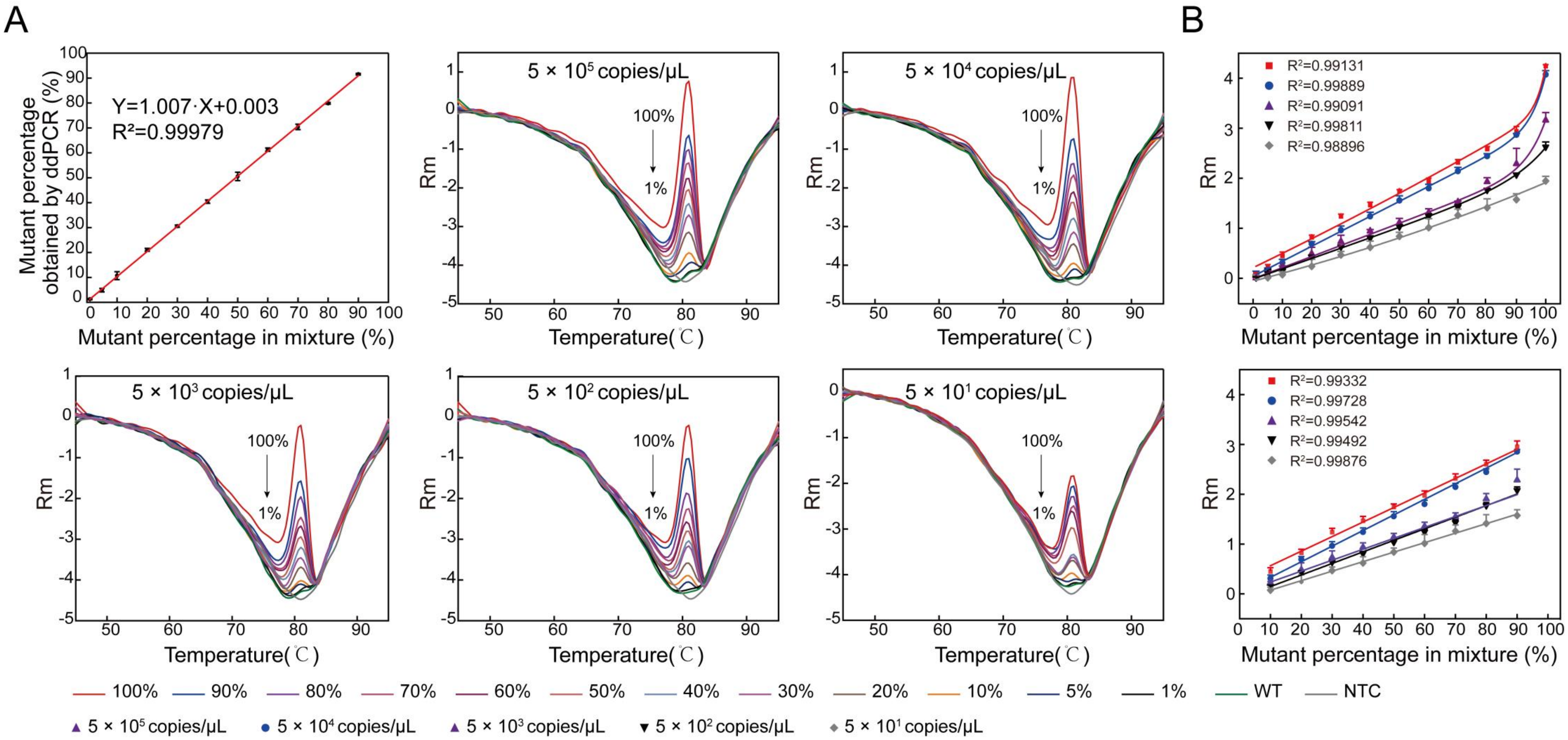

Fig S7 MeltArray analysis was conducted at five different genomic concentrations, using *gyrA* D94G as an example. (A) Melting curve analysis for detecting *gyrA* D94G at concentrations of 5×10^5 copies/µL, 5×10^4 copies/µL, 5×10^3 copies/µL, 5×10^2 copies/µL, and 5×10^1 copies/µL. (B) Analysis of the exponential and linear relationships between the Rm and MUT% across the five genomic concentrations.
